# Breastfeeding frequency and incidence of type 2 diabetes among women with previous gestational diabetes compared to those without: a historical cohort study in the UK

**DOI:** 10.1186/s13006-024-00679-0

**Published:** 2024-10-17

**Authors:** Claire Eades, Pat Hoddinott, Dawn Cameron, Josie Evans

**Affiliations:** 1 Faculty of Health Sciences and Sport, University of Stirling, Stirling, FK9 4LA Scotland; 2 School of Health and Life Sciences, University of West of Scotland, Lanarkshire Campus, Ayr, G72 0LH Scotland; 3 Public Health Scotland, Edinburgh, Scotland

**Keywords:** Gestational diabetes, Breastfeeding, Prevention, Type 2 diabetes

## Abstract

**Background:**

There is a growing body of research to suggest that women with gestational diabetes are less likely to initiate and continue breastfeeding than those who have not had however findings are mixed. There is limited research in the UK assessing the frequency of breastfeeding in women with gestational diabetes, none reporting the association of breastfeeding with incidence of type 2 diabetes and existing research has not adequately adjusted for potential confounders. This study aims to assess frequency of breastfeeding among women with gestational diabetes compared to those without, and to explore how breastfeeding influences risk of future type 2 diabetes in women with gestational diabetes while adjusting for known confounders.

**Methods:**

Historical cohort study using routinely collected health care data from Fife and Tayside Health Boards, Scotland, UK including all women diagnosed with gestational diabetes between 1993 and 2015 and a matched comparator cohort (*n* = 4,968). Women with gestational diabetes were followed up until a diagnosis of type 2 diabetes, the end of the study, or date of death. Multinomial logistic regression was used to estimate odds ratios for breastfeeding for the whole sample and the association between breastfeeding and development of type 2 diabetes in women with gestational diabetes was assessed by Cox regression.

**Results:**

Women with a diagnosis of gestational diabetes, who were younger, overweight/obese or living in the most deprived areas were significantly less likely to exclusively breastfeed for a duration of longer than eight weeks. Risk of developing type 2 diabetes among women with gestational diabetes was significantly higher for those who exclusively breastfed less than 8 weeks, lived in the most deprived areas or had a family history of diabetes.

**Conclusions:**

This study confirms the important role of a short duration of exclusive breastfeeding in protecting women with gestational diabetes against type 2 diabetes but highlights the challenges to breastfeeding in this group. Interventions are needed to support breastfeeding among women with gestational diabetes that are acceptable to younger, overweight/obese women living in deprived areas.

## Introduction

Gestational Diabetes Mellitus (GDM) affects around 5% of women in Europe and is defined as glucose intolerance that is first diagnosed in pregnancy [1]. GDM increases the risk of complications for the mother and child, including a seven-fold increased risk of type 2 diabetes (T2D) among women who have had GDM compared to those without [2]. Rates of GDM have increased over recent years with one Canadian study reporting that prevalence almost doubled in a decade from 4.6% in 2006 to 8.2% in 2016 [3] and the international diabetes federation reporting that around 21 million live births were complicated by GDM in 2021 [4]. The benefits of breastfeeding for both women and their babies are well documented, and it has been suggested that there are specific benefits for women with pregnancies complicated by GDM including a reduction in the incidence of T2D [5–7]. There is a growing body of research to suggest that women with GDM are less likely to initiate and continue breastfeeding than those who have not had GDM [6], however findings are mixed.

Only one study has been identified in the UK that assesses frequency of breastfeeding in a small group of women with GDM and a comparison group [8], and none report the association of breastfeeding with incidence of T2D. The study by Logan et al. [8] with 86 infants did not find any significant difference in exclusive or predominant breastfeeding at 8 to 12 weeks between women with GDM and those without. This study and other non-UK studies have not adequately adjusted for potential confounders such as obesity and insulin use in pregnancy when assessing the relationship between breastfeeding and T2D in women with GDM [9]. It is important to understand these issues in the UK context, where the prevalence of breastfeeding is relatively low for developed countries [10]. In the most recent Scottish infant feeding survey for 2021/2022 66% of babies were breastfed at birth, 37% were being exclusively breastfed at 2 weeks and 32% at 8 weeks in 2021 [11]. An understanding of breastfeeding frequency among women with GDM could inform intervention development and evaluation for these women. This study aims to use routinely collected UK health care data to investigate a historical cohort to assess the frequency of breastfeeding in women with GDM and to assess how exclusive breastfeeding influences the risk of T2D among women with GDM with adjustment for potential confounders.

### Methods

#### Design

Historical cohort study using routinely collected, anonymised health care data for the population of pregnant women in the Fife and Tayside Health Boards in Scotland, United Kingdom (UK). Data were provided by the Health Informatics Centre (HIC) of the University of Dundee who have developed the record linkage of routinely collected health care datasets. Data held by HIC are anonymised to ensure confidentiality and meet data protection legislation.

#### Population

The study population was all women with a diagnosis of GDM in Tayside and Fife Health Boards, Scotland, between September 1993 to May 2015 (*n* = 2499) and a matched comparator cohort of women giving birth during the same time period who did not have GDM (*n* = 2499). NHS Tayside health board has a current population of approximately 416,000 and the neighbouring NHS Fife health board has a population of approximately 374,000 [12]. A validated population-based diabetes clinical information system, SCI-DC, was used to identify women diagnosed with GDM during the study period. The original SCI-DC database for Tayside had 95% sensitivity at identifying people with diabetes but only a small subset of women with GDM were included in the validation study [13].

Data from SCI-DC were then linked to SMR02, which is the maternity inpatient and day case dataset in Scotland, to provide demographic and clinical information for the study population such as mother’s age, deprivation category, Body Mass Index (BMI) at first antenatal appointment, and insulin use. Women with serious maternal health problems (heart disease, alcohol dependence, syndrome/alcoholism, substance abuse, HIV and hepatitis B; *n* = 12) or neonatal death/stillbirth complications (*n* = 3) were excluded from the study leaving a final population of 2484 women with GDM. Women without GDM were selected from the SMR02 dataset and matched to women with GDM based on their Scottish Index of Multiple Deprivation (SIMD) quintile. SIMD is an area-based measure of relative deprivation calculated using 30 indicators across seven domains: income, employment, health, access to services, crime and housing [14]. Where possible women with GDM were also matched to those without GDM according to parity. Exact matches on parity were made for 63% of pairs of women (*n* = 1564) and 19% of pairs (*n* = 477) were matched more generally on nulliparity and parity. It was not possible to make a match on parity for 16% of pairs (*n* = 397) or for 2% of pairs (*n* = 46) who had missing parity data. The Child Health dataset provided data on breastfeeding status (exclusive breastfeeding, mixed, bottle, or other) for the first feed after birth, upon discharge following birth, at visit from the midwife (between discharge and 10 days postpartum), at the first health visitor visit (around 2 weeks postpartum) and at the 6 to 8 week visit from the health visitor. Bottle feeding in the present study refers to formula fed via bottle. Missing data on breastfeeding status in the Child Health dataset were supplemented with data from SMR02 where it was available for the first feed after birth and feeding status at discharge. Women with GDM and those without were followed up for a diagnosis of T2D using SCI-DC until a date of diagnosis of T2D was made, or until the end of the study or date of death. A diagnosis of T2D in SCI-DC was made using the World Health Organisation (WHO) criteria, but the precise glucose levels used depended upon the criteria in use at the time of diagnosis. Similarly, diagnoses of GDM were made based on clinical guidance in use at the time of the study.

#### Analysis

Differences in the demographic characteristics and feeding status (exclusive breastfeeding, mixed feeding, bottle, or other) of women with GDM and those without were explored using chi-square tests of independence. Multinomial logistic regression was used to estimate crude and adjusted odds ratios for breastfeeding for the whole sample with exclusive breastfeeding duration as the dependent variable and diagnosis of GDM, maternal age, BMI, parity, deprivation category and baby birthweight as predictor variables. The association between breastfeeding and development of T2D in women with GDM was assessed by univariate and multivariate Cox regression from which hazard ratios (HRs) and 95% CIs were calculated. Breastfeeding duration, maternal age, parity, deprivation category, BMI, family history of T2D were entered as independent variables, with diagnosis of T2D as the dependent variable. Statistical analyses were carried out using SPSS for Windows version 25.

## Results

### Characteristics of sample

The characteristics of women and their babies are outlined in Table 1. Women with GDM were significantly older, more deprived and had higher BMIs and their babies were more likely to have a lower APGAR score and to be born at an earlier gestation than those whose mothers did not have GDM.

**Table 1:** Characteristics of women in the study sample and comparison between groups

Variables	GDM group Total *n* = 2,484	No GDM group Total *n* = 2,484
***Maternal Data***		
**Age at delivery**	*n* = 2,460	*n* = 2,484
mean (SD)	31.2 (6.8)	27.3 (7.0)
t(df)	19.9 (4942)*	
**Parity**	*n* = 2,440	*n* = 2,475
Nulliparous	40.6%	41.6%
Multiparous	59.4%	58.4%
χ2 (df)	0.5 (1)	
**BMI**	*n* = 1,690	*N* = 1,489
mean (SD)	33.8 (0.2)	25.7 (0.1)
t(df)	34.1 (3177)*	
**SIMD quintile**	*n* = 2,181	*n* = 2,237
1 (most deprived)	28.6%	24.5%
2	22.4%	20.8%
3	17.7%	20.9%
4	16.1%	17.2%
5 (least deprived)	15.4%	16.6%
χ2 (df)	15.8 (4)*	
***Newborn Data***		
**Birth weight in grams**	*n* = 1,996	*n* = 2,482
mean (SD)	3421 (588)	3417 (501.5)
t(df)	0.3 (4476)	
**Gestational age (weeks)**	*n* = 1,695	*n* = 2,016
mean (SD)	38.3 (2.1)	39.8 (1.8)
t(df)	19.8 (4942)*	
**Gender (%)**	*n* = 1,996	*n* = 2,484
Male	52.9%	51.6%
Female	47.1%	48.4%
χ2 (df)	0.7 (1)	
**Baby APGAR**	*n* = 1,962	2,376
0–7	3.7%	1.6%
8–10	96.3%	98.4%
χ2 (df)	19.4 (1)*	

### Availability of feeding data

Records of how women fed their babies were not complete with data missing to different extents at all five time points. The most complete data on feeding were found at discharge with data available for 84.7% of women in the study, followed by birth (78.8%), midwife visit (68.9%), 8-week health visitor visit (59.5%) and the 2 week health visitor visit (58.5%).

### Breastfeeding frequency

**Fig. 1:**
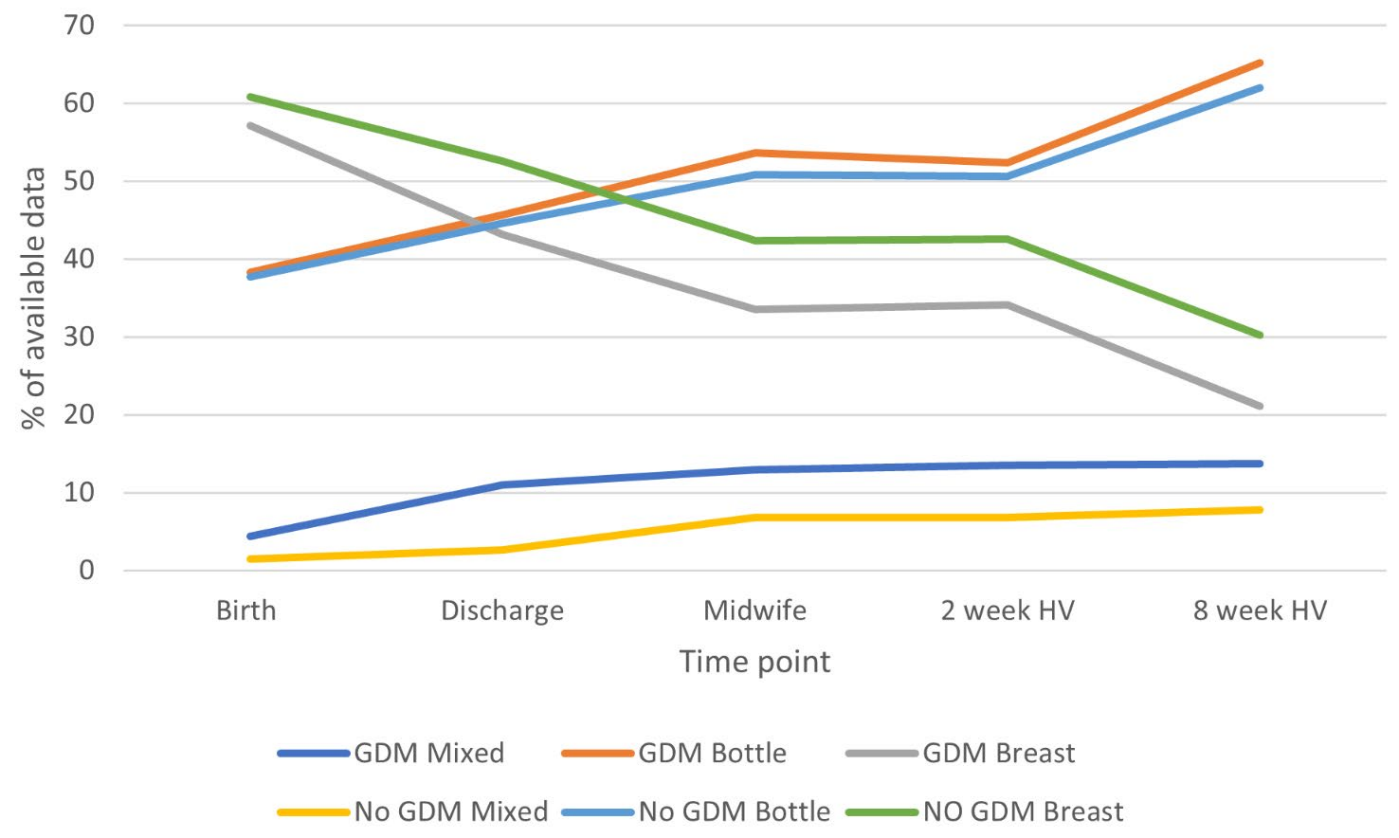
Percentage of women with GDM and without who breast, mixed or bottle fed at five time points

Figure 1 shows the percentage of women with GDM and without GDM who were bottle feeding, breastfeeding and mixed feeding (bottle and breast) at the five different time points (birth, discharge, midwife visit, 2 weeks and 8 weeks). For all women, breastfeeding frequency decreases steadily from birth to 8 weeks, while bottle feeding increases. At birth 57.1% of women with GDM and 60.8% of women without GDM breastfed their baby compared to only 21.1% of women with GDM and 30.2% of women without GDM at 8 weeks. The greatest increase in mixed feeding (bottle and breast) was observed between birth and discharge for women with GDM, and between birth and the midwife visit for women without GDM. Mixed feeding then remains relatively stable for both groups. Association between GDM status and feeding type as assessed by Chi-square test of independence was significant at birth (χ2 = 30.7 (3), *p* < 0.001), discharge (χ2 = 124.6 (3), *p* < 0.001), midwife, 2 weeks (χ2 = 23.1 (3), *p* < 0.001) and 8 weeks (χ2 = 32.7 (3), *p* < 0.05). Women with GDM had a lower frequency of exclusive breastfeeding and higher frequency of bottle and mixed feeding than those without GDM.

### Factors influencing breastfeeding

A multinomial logistic regression was performed to assess the relationship between GDM status and the duration of exclusive breastfeeding (no exclusive breastfeeding, exclusively breastfeeding at birth only, exclusively breastfeeding until midwife visit and exclusively breastfeeding at 2 weeks or 8 weeks) with adjustment for independent variables that were potential confounding factors (maternal age, parity, deprivation category, BMI and baby birthweight). Adjustment for independent variables significantly improved the fit of the model (X^2^(45) = 9322.07, *p* < 0.001) with all independent variables having a significant impact on duration of exclusive breastfeeding (*p* < 0.001) versus no exclusive breastfeeding. The results of the multinomial regression in Table 2 show that women with a diagnosis of GDM were significantly less likely to breastfeed at later time points compared to women without GDM with odds ratios of 0.26 (95% CI 0.08–0.80) for breastfeeding at the midwife visit, 0.61 (95% CI 0.42–0.88) for breastfeeding until 2 weeks and 0.60 (95% CI 0.44–0.81) for breastfeeding at 8 weeks. Older maternal age, nulliparty, living in an area with a lower deprivation quintile, lower BMI, and higher birthweight were all significant independent predictors of breastfeeding.

**Table 2:** Odds ratios for different durations of exclusive breastfeeding from a multinomial logistic regression in the whole sample with GDM status, maternal age, parity, deprivation category, BMI and baby birthweight entered as predictor variables

	Odds Ratio of breastfeeding at each time points									
	Birth Exp (B) (95% CI)	**p**	Discharge Exp (B) (95% CI)	**p**	Midwife visit Exp (B) (95% CI)	**p**	2 weeks Exp (B) (95% CI)	**p**	8 weeks Exp (B) (95% CI)	**p**
**GDM status (total ** ***n *** **= 2,866)**										
GDM (*n* = 1,649)	1.36 (0.99–1.86)	0.059	1.12 (0.89–1.41)	0.331	0.26 (0.08–0.80)	0.019	0.61 (0.42–0.88)	0.008	0.60 (0.44–0.81)	0.001
No GDM (*n* = 1,728)	1.00									
**Maternal age at delivery (total ** ***n*** ** = 4,944)**	1.04 (1.02–1.07)	< 0.001	1.05 (1.03–1.07)	< 0.001	1.02 (0.95–1.10)	0.594	1.04 (1.02–1.07)	0.003	1.09 (1.07–1.11)	< 0.001
**Parity (total ** ***n*** ** = 2,443)**										
Nulliparous (*n* = 1,339)	2.24 (1.71–2.94)	< 0.001	1.49 (1.21–1.83)	< 0.001	3.28 (1.33–8.09)	0.010	2.4 (1.75–3.38)	< 0.001	1.73 (1.31–2.30)	< 0.001
Multiparous (*n* = 2,038)	1.00									
**SIMD quintile (total ** ***n*** ** = 2,181)**										
1 (most deprived, *n* = 890)	0.54 (0.35–0.84)	0.006	0.32 (0.24–0.44)	< 0.001	0.51 (0.14–1.89)	0.310	0.38 (0.23–0.61)	< 0.001	0.30 (0.20–0.46)	< 0.001
2 (*n* = 720)	0.64 (0.41-1.00)	0.052	0.42 (0.31–0.58)	< 0.001	0.46 (0.11–1.93)	0.289	0.48 (0.29–0.79)	0.004	0.36 (0.23–0.55)	< 0.001
3 (*n* = 653)	0.75 (0.47–1.18)	0.210	0.52 (0.38–0.73)	< 0.001	0.55 (0.13–2.29)	0.412	0.39 (0.23–0.69)	< 0.001	0.47 (0.31–0.72)	< 0.001
4 (*n* = 562)	0.94 (0.58–1.51)	0.793	0.58 (0.41–0.82)	0.002	0.80 (0.20–3.31)	0.761	0.80 (0.48–1.33)	0.376	0.67 (0.44–1.03)	0.065
5 (least deprived, *n* = 552)	1.00									
**BMI (** ***n*** ** = 2,702)**										
Normal (*n* = 834)	0.69 (0.47-1.00)	0.052	1.64 (1.26–2.12)	< 0.001	1.13 (0.34–3.7)	0.846	1.04 (0.67–1.60)	0.876	2.98 (2.01–4.30)	< 0.001
Overweight (*n* = 651)	0.95 (0.67–1.33)	0.746	1.45 (1.13–1.88)	0.004	1.95 (0.63–6.10)	0.250	1.66 (1.12–2.48)	0.012	2.69 (1.87–3.86)	< 0.001
Obese (*n* = 1217)	1.00									
**Birthweight baby kg (** ***n*** ** = 4,478)**	1.04 (1.02–1.07)	0.001	1.04 (1.02–1.06)	< 0.001	1.03 (0.95–1.12)	0.486	1.31 (1.00-1.06)	0.047	1.07 (1.05–1.10)	< 0.001

### Breastfeeding and T2D incidence

Among the 2,484 women with a diagnosis of GDM, 16.0% (*n* = 395) developed T2D during the study period with a mean time of 77 months/6.4 years between GDM and T2D diagnoses. Table 3 shows the results of univariate and multivariate cox regression assessing the hazard ratio for developing T2D among women with GDM according to duration of exclusive breastfeeding, maternal age, parity, deprivation category, BMI, family history of diabetes, medication or insulin treatment in pregnancy. Women who did not exclusively breastfeed at all or who only exclusively breastfed at birth were at a significantly increased risk of developing T2D compared to those who were exclusively breastfeeding at 8 weeks with hazard ratios of 1.44 (95% CI 0.98–2.12) and 1.76 (95% CI 1.13–2.74) respectively. In the multivariate analyses, only the increased risk for exclusively breastfeeding at birth remained significant for developing T2D with a hazard ratio of 2.2 (95% CI 1.21-4.00) compared to those who were still exclusively breastfeeding at 8 weeks. In both univariate and multivariate analyses women living in the more deprived areas (SIMD category 1 and 2) were significantly more likely to develop T2D compared to those in the least deprived areas (SIMD category 5) with hazard ratios of 2.69 (95% CI 1.56–4.62) and 2.36 (95% CI 1.34–4.15) respectively in the multivariate analysis. Family history of diabetes significantly increased risk of T2D in univariate and multivariate analyses with a hazard ratio of 1.77 (95% CI 1.29–2.42) compared to those without a family history in the multivariate analysis. Maternal age, parity and obesity did not impact on risk of developing T2D in either univariate or multivariate analyses.

**Table 3:** Hazard ratio of developing T2D in women with GDM according to duration of exclusive breastfeeding, maternal age, parity, deprivation category, BMI and family history

	Univariate				Multivariate	
	No. (%) progressing to T2D*	Mean time to progress (months)	Hazard ratio (95% CI)	*P* value	Hazard ratio	*P* value
**Latest point baby exclusively breastfeeding (total ** ***n*** ** = 1, 378)**						
None (*n* = 585)	154 (26.3)	67.6	1.44 (0.98–2.12)	0.065	1.49 (0.86–2.58)	0.151
At Birth (*n* = 209)	56 (26.8)	54.0	1.76 (1.13–2.74)	0.012	2.20 (1.21-4.00)	0.010
At Discharge (*n* = 399)	74 (18.5)	68.5	1.20 (0.79–1.83)	0.399	1.70 (0.95–3.03)	0.075
At midwife visit (*n* = 3)	0 (0)	0	0.0 (0.0–0.0)	0.935	0.0 (0.0–0.0)	0.958
At 2 weeks (*n* = 71)	26 (36.6)	73.8	1.22 (0.72–2.05)	0.459	1.81 (0.91–3.60)	0.089
At 8 weeks (*n* = 111)	31 (27.9)	77.6	1		1	
**Maternal age at delivery (total ** ***n*** ** = 2,458)**						
< 20 (*n* = 89)	14 (15.7)	69.5	1		1	
20–24 (*n* = 278)	46 (16.5)	61.6	1.11 (0.61–2.03)	0.723	2.87 (0.39–21.15)	0.302
25–34 (*n* = 1322)	221 (16.7)	73.0	1.04 (0.61–1.78)	0.889	1.93 (0.27–13.96)	0.515
35 and over (*n* = 769)	108 (14.0)	76.2	0.95 (0.54–1.65)	0.847	1.92 (0.26–14.05)	0.520
**Parity (total ** ***n*** ** = 2, 443)**						
Nulliparous (*n* = 991)	159 (16.0)	73.0	1		1	
Multiparous (*n* = 1452)	230 (15.8)	71.7	1.06(0.87–1.3)	0.548	1.03 (0.77–1.37)	0.850
**SIMD quintile (** ***n*** ** = 1, 378)**						
1 (most deprived, *n* = 385)	116 (18.6)	65.2	1.78 (1.26–2.51)	< 0.001	2.69 (1.56–4.62)	< 0.001
2 (*n* = 306)	80 (16.3)	70.4	1.55 (1.08–2.24)	0.017	2.36 (1.34–4.15)	0.003
3 (*n* = 250)	59 (15.3)	60.2	1.51 (1.03–2.23)	0.036	1.81 (1.0-3.28)	0.051
4 (*n* = 239)	54 (15.3)	82.0	1.18 (0.80–1.75)	0.404	1.75 (0.98–3.14)	0.061
5 (least deprived, *n* = 198)	46 (13.7)	98.5	1		1	
**BMI (total ** ***n*** ** = 1,686)**						
Normal (210)	29 (13.8)	80	1		1	
Overweight (332)	52 (15.7)	86.3	1.08 (0.68–1.70)	0.747	1.10 (0.65–1.85)	0.727
Obese (1135)	187 (16.5)	72.3	1.13 (0.76–1.67)	0.549	1.15 (0.73–1.79)	0.550
**Family History of Diabetes (total** *** n*** ** = 2, 484)**						
Yes (341)	122 (35.8)	88.4	1.88 (1.51–2.33)	< 0.001	1.77 (1.29–2.42)	< 0.001
No/Not recorded (2,143)	273 (12.7)	65.6	1		1	

*There was missing data for all variables except family history. Of the 395 who developed T2D, data on feeding were available for 341, data on maternal age and parity were available for 389, data on SIMD were available for 355, and data on BMI were available for 268

## Discussion

### Main findings

In this study we found that the likelihood of a longer duration of exclusive breastfeeding was significantly lower among women with a diagnosis of GDM, who were younger, overweight or obese or living in the most deprived areas. Exclusive breastfeeding frequency at birth was broadly similar in women with GDM and those without, but women with GDM were significantly less likely to continue breastfeeding up to 8 weeks compared to those without GDM. Although initiation of breastfeeding appeared similar in both groups, a steeper increase in bottle feeding was seen over time in women with GDM. The risk of developing T2D among women with GDM was higher for those who exclusively breastfed for less than eight weeks, or lived in the most deprived areas or had a family history of diabetes.

### Strengths and limitations

To the best of our knowledge this study is the first to investigate breastfeeding frequency and incidence of T2D in the UK. Using routinely collected data provides a larger sample than previous research on this topic [9] and allows for adjustment for known confounders, such as obesity and family history, in the relationship between breastfeeding and T2D incidence among women who have had GDM. This study confirms the important role of breastfeeding in reducing the risk of developing T2D after a diagnosis of GDM in pregnancy after adjustment for known confounders such as obesity. It is also one of few studies to explore and confirm the role of deprivation on the progression to T2D among women with GDM. The study uses an extensively tested and highly accurate clinical information system to provide information about diagnoses of GDM and T2D [13]. Furthermore, the region in which the study was carried out is broadly representative of the population of Scotland and the results are likely to be generalizable to the UK as breastfeeding rates and trajectories over time are similar [15, 16]. However, there was a relatively high level of missing data for several variables of interest which may have limited the ability of the study to detect relationships and associations. Data was available on age for the whole sample and for most of the sample (89.5%) for birthweight of the baby. Data on feeding, parity, deprivation category and BMI were only available for 67.6%, 48.9%, and 54.2% of the sample respectively. Data came from only two of the 14 Health Boards in Scotland. Another limitation is that we do not know the specific diagnostic criteria used for diagnoses of GDM due to transitions in diagnostic criteria during the study period and local differences in adoption of these. The breastfeeding rates reported in the present study for women without GDM are similar to those reported by Public Health Scotland in their most recent annual infant feeding statistics for 2022/2023 [11]. Given that breastfeeding rates in Scotland have generally increased over time [11] and that the data in this study is over 10 years old at time of publication, this may mean that the women who had breastfeeding status recorded in this study were those who were more likely to breastfeed. It is also possible that changes in clinical care and support for women to breastfeed may mean that our findings may not be entirely transferable to the present-day context.

### Interpretation

This study contributes to a growing body of research to suggest that women with GDM are less likely to initiate and continue breastfeeding than those who have not had GDM [5]. Possible explanations for lower breastfeeding rates among women with GDM have included delays in milk production, higher rates of caesarean section delivery, lower Apgar scores and higher rates of admission to neonatal intensive care units among women with GDM [17, 18]. Furthermore, obesity may also cause difficulties in latching [19]. We found that a diagnosis of GDM was associated with significantly reduced likelihood of breastfeeding at eight weeks even after adjusting for confounding variables such as BMI, deprivation, maternal age, and birthweight of the baby. This suggests that there may be unique challenges to breastfeeding for women with a diagnosis of GDM that cannot only be explained by increased prevalence of obesity.

Although there is a body of research exploring the lower frequency of breastfeeding in obese women and the reasons for this, these studies generally exclude women with GDM or adjust analysis according to diagnosis of GDM [20]. An integrative review aiming to identify factors that positively influence breastfeeding among women with GDM while they are in hospital identified that while many of the reasons women with GDM introduced formula milk were similar to those of the general population, specific concerns around their baby’s hypoglycaemia, delayed lactogenesis and low milk supply highlight a need for tailored support for women with GDM [21]. There may therefore be scope to improve breastfeeding outcomes in women with GDM but they may need tailored support [22]. Our study highlights that women with GDM, who are younger, overweight or obese and living in the most deprived areas least likely to exclusively breastfeed at 8 weeks suggesting that these women in particular should be targeted for support.

We found that the risk of developing T2D among women with GDM was significantly higher for those who exclusively breastfed for less than eight weeks, or lived in the most deprived areas or had a family history of diabetes. Our study only included data on breastfeeding up to eight weeks following delivery as data on longer terms feeding outcomes was not available but a meta-analysis of five studies by Tanase-Nakao et al. [9] reported that longer duration of breastfeeding (greater than 4 to 12 weeks) had a significant association with lower risk of T2D compared to shorter duration (less than 4 to 12 weeks). Our study supports the benefits of even a short period of breastfeeding on T2D risk which is important as this may be a more achievable goal for many women.

In our study, the risk of developing T2D among women with GDM was significantly higher for those who exclusively breastfed for a shorter duration, lived in the most deprived areas and had a family history of diabetes when parity, maternal age and BMI were adjusted for. Similarly, Zieglar et al. [23], Urs and Chandwani [24] and Ley [5] reported that breastfeeding was protective against T2D even when BMI, maternal age and parity were taken into account. However, unlike the present study, Zieglar et al. [23] reported that BMI was also a significant predictor of T2D. These differences in the findings on BMI as a risk factor may be due to inclusion of deprivation as a covariate in our study which remained a significant predictor of T2D in multivariate analyses. People living in the most deprived areas in Scotland are more likely to overweight or obese than those in the least deprived areas [25]. The findings of this study suggest that the relationship between BMI, deprivation and incidence of T2D following GDM warrants further investigation to see if our findings are replicated.

## Conclusions

This study found that even a relatively short duration of exclusive breastfeeding protects women with GDM against future risk of T2D. There appear to be unique challenges facing women with GDM in breastfeeding that cannot solely be accounted for by the confounding factors of obesity, parity and maternal age suggesting that women with GDM are likely to need tailored support to breastfeed. Interventions are needed that are acceptable to women with GDM who live in the most deprived areas. Research is needed to understand the unique challenges faced by women with GDM living in deprived areas to allow appropriate support to be identified.

## Data Availability

The data that support the findings of this study are available from the Health Informatics Centre (University of Dundee) but restrictions apply to the availability of these data, which were used under license for the current study, and so are not publicly available. Data are however available from the authors upon reasonable request and with permission of the Health Informatics Centre.

## Abbreviations

BMI: Body Mass Index
GDM: Gestational Diabetes Mellitus
HIC: Health Informatics Centre
SIMD: Scottish Index Multiple Deprivation
SMR02: Scottish Morbidity Record 02
T2D: Type 2 Diabetes
WHO: World Health Organisation
